# Supplementary material for: HTNV infection of CD8+ T cells is associated with disease progression in HFRS patients
Source: Commun Biol. 2021 Jun 2;4:652. doi: 10.1038/s42003-021-02182-2 (PMC8173013; doi:10.1038/s42003-021-02182-2)
Supplement: Supplementary file 2 — Supplementary Information [file 42003_2021_2182_MOESM2_ESM.pdf]

Supplementary Information:

HTNV infection of CD8$^+$ T cells is associated with disease progression in HFRS patients

Rongrong Liu[1†], Ruixue Ma[1†], Ziyu Liu[1†], Haifeng Hu[2], Jiayi Shu[3], Peizhen Hu[4], Junjun Kang[5], Yusi Zhang[5], Mingwei Han[5], Xiaoxiao Zhang[1], Yiting Zheng[5], Qikang Ying[1], Shiyuan Hou[1], Wenqiu Wang[5], Fang Wang[1], Ning Cheng[6] Yan Zhuang[2], Jianqi Lian[2], Xia Jin[3]*, Xingan Wu[1]*

[1]Department of Microbiology, School of Basic Medicine, Fourth Military Medical University, Xi'an, China

[2]Department of Infective Diseases, Tangdu Hospital, Fourth Military Medical University, Xi'an, China

[3]Shanghai Public Health Clinical Center, Fudan University, Shanghai, China

[4]State Key Laboratory of Cancer Biology, Department of Pathology, Xijing Hospital and School of Basic Medicine, Fourth Military Medical University, Xi'an, China

[5]School of Basic Medicine, Fourth Military Medical University, Xi'an, China

[6]Department of Otolaryngology-Head and Neck Surgery, University of California San Francisco, San Francisco, California 94115, USA.

* Corresponding authors :

wuxingan@fmmu.edu.cn, or jinxia@shphc.org.cn;

† These authors contributed equally to the work

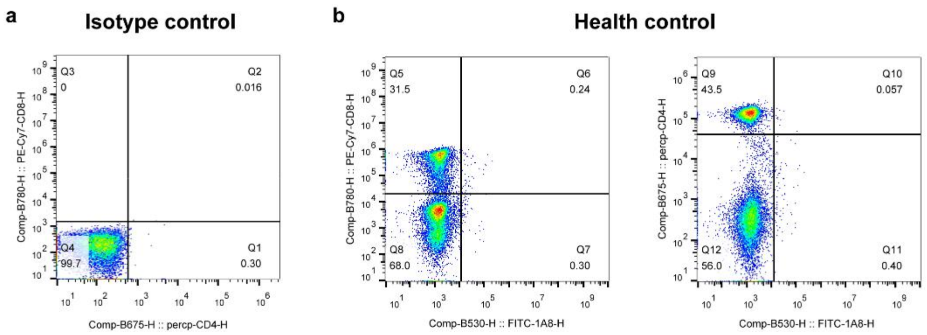

Supplementary Figure 1. Representative FACS plots of isotype control and HTNV-NP staining from uninfected (healthy) donors. **a)** FITC mouse IgG1, κ antibody served as an isotype control of FITC-mAb 1A8 used in this study（n=3）; **b)** PBMCs isolated from healthy donors were stained with PE/Cy7-CD8 or Percp-CD4 and FITC-mAb 1A8 served as normal control（n=3）.

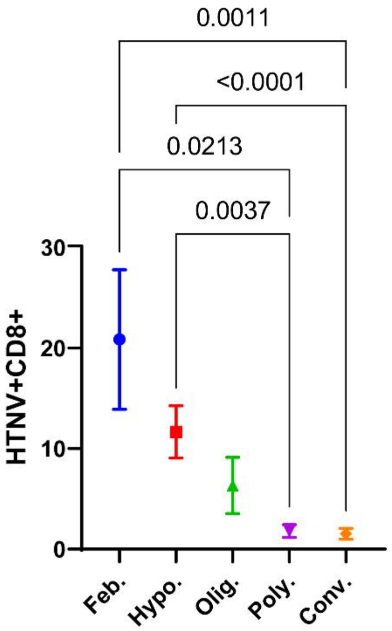

Supplementary Figure 2. Comparison between the frequency of HTNV-infected $CD8^+$ T cells according to the disease stage. Feb. is short for febrile（n=14）; Hypo. is short for hypotensive（n=9）; Olig. is short for oliguric（n=37）; Poly. is short for polyuric（n=24）; Con. is short for convalescent（n=32）. Values are expressed as the median (IQR). One-way ANOVA with a post hoc Tukey test was used for multiple comparisons.

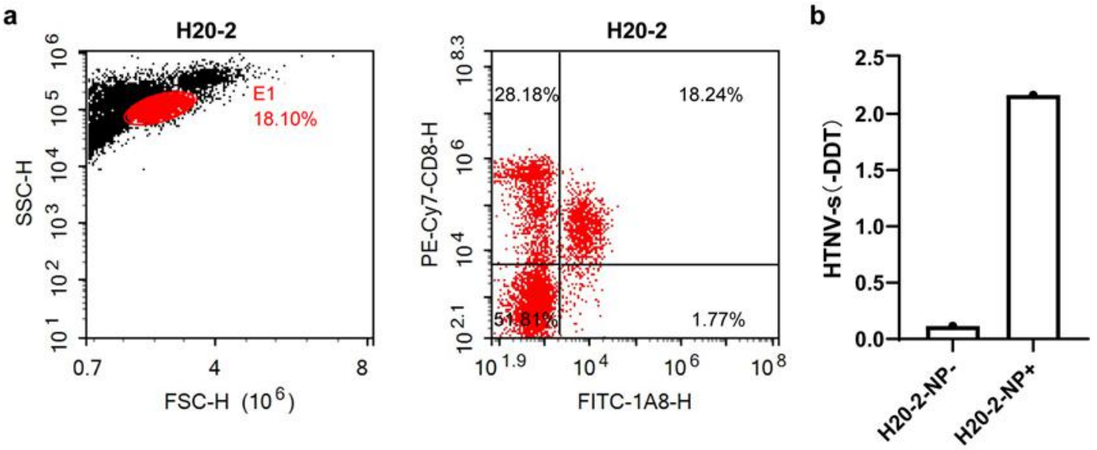

Supplementary Figure 3. Identification of HTNV infection of CD8$^+$ T cells (NP$^+$ CD8$^+$) and CD8$^+$ T cells (NP$^-$ CD8$^+$) among PBMCs from an HFRS patient (H20-2). **a)** Gating strategy for defining major subsets of lymphocytes and esentative FACS plots of PBMCs stained with PE/Cy7-CD8 and FITC-mAb 1A8. b**)** HTNV-*s* mRNA expression were measured in the two populations through qPCR on freshly isolated patient H20-2.

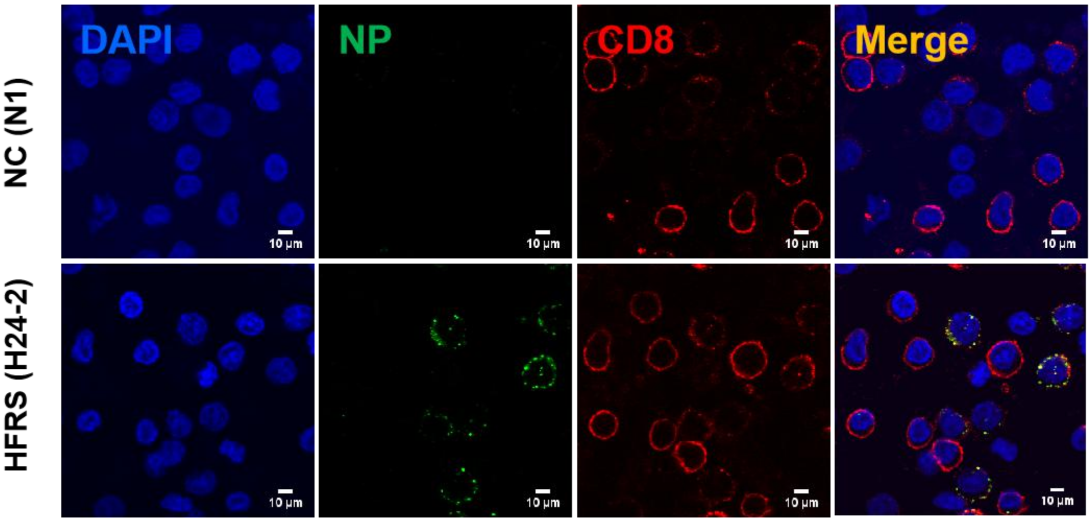

Supplementary Figure 4. Identification of HTNV infection of CD8$^+$ T cells (NP$^+$ CD8$^+$) among PBMCs from an HFRS patient (H24-2). PBMCs were stained with HTNV NP (green), CD8 (red), and the nucleus with DAPI (blue). HTNV NP or CD8 single-positive cells, as well as NP$^+$ CD8$^+$ double-positive (orange) cells, are shown.

A normal control sample from an uninfected subject (N1) was included for comparison.

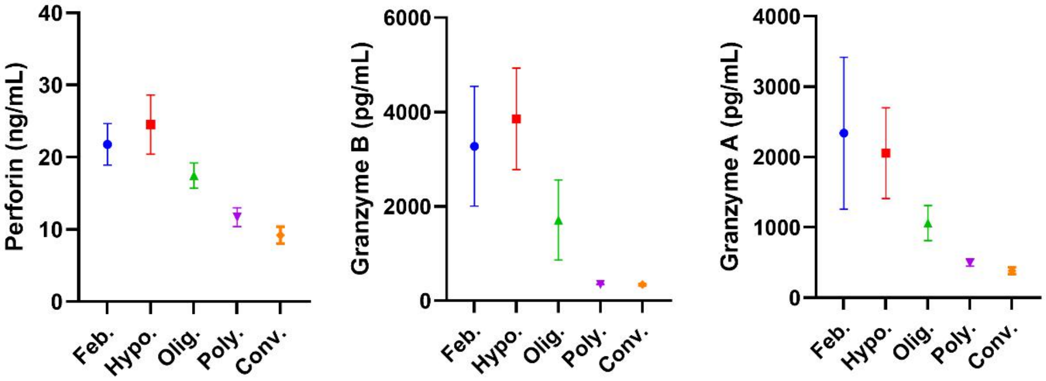

Supplementary Figure 5. The amounts of granzyme A, granzyme B, and perforin in HFRS patient’s plasma were measured by ELISA according to the disease stage. Values are expressed as the median (IQR) from 3 independent experiments. One-way ANOVA with a post hoc Tukey test was used for multiple comparisons. Feb. is short for febrile（n=14）; Hypo. is short for hypotensive（n=10）; Olig. is short for oliguric（n=37）; Poly. is short for polyuric（n=35）; Con. is short for convalescent（n=24）.

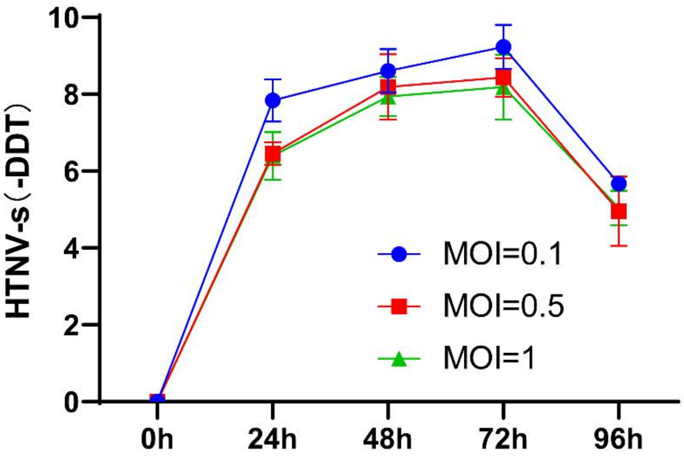

Supplementary Figure 6. T cells infected by HTNV of different MOI. At 0, 24, 48, 72, and 96 h post-infection of HTNV (MOI=0.1,0.5,1), viral loads were measured by

the detection of the HTNV *s* gene segment using quantitative real-time PCR, and β-actin was used as a housekeeping gene for normalization（n=5）.
