# Supplementary material for: HTNV infection of CD8+ T cells is associated with disease progression in HFRS patients
Source: Commun Biol. 2021 Jun 2;4:652. doi: 10.1038/s42003-021-02182-2 (PMC8173013; doi:10.1038/s42003-021-02182-2)
Supplement: Supplementary file 3 — Description of Additional Supplementary Files [file 42003_2021_2182_MOESM3_ESM.pdf]

Description of Additional Supplementary Files

File name: Supplementary Data 1

Description: Source data for all figures are provided with the paper
