# Supplementary material for: HTNV infection of CD8+ T cells is associated with disease progression in HFRS patients
Source: Commun Biol. 2021 Jun 2;4:652. doi: 10.1038/s42003-021-02182-2 (PMC8173013; doi:10.1038/s42003-021-02182-2)
Supplement: Supplementary file 5 — Reporting Summary [file 42003_2021_2182_MOESM5_ESM.pdf]

nature research

Corresponding author(s): Xing-an Wu and Xia Jin

Last updated by author(s): Apr 17, 2021

Reporting Summary

Nature Research wishes to improve the reproducibility of the work that we publish. This form provides structure for consistency and transparency in reporting. For further information on Nature Research policies, see our Editorial Policies and the Editorial Policy Checklist.

Statistics

For all statistical analyses, confirm that the following items are present in the figure legend, table legend, main text, or Methods section.

n/a	Confirmed	
☐	☒	The exact sample size (*n*) for each experimental group/condition, given as a discrete number and unit of measurement
☐	☒	A statement on whether measurements were taken from distinct samples or whether the same sample was measured repeatedly
☐	☒	The statistical test(s) used AND whether they are one- or two-sided<br>*Only common tests should be described solely by name; describe more complex techniques in the Methods section.*
☐	☒	A description of all covariates tested
☒	☐	A description of any assumptions or corrections, such as tests of normality and adjustment for multiple comparisons
☐	☒	A full description of the statistical parameters including central tendency (e.g. means) or other basic estimates (e.g. regression coefficient) AND variation (e.g. standard deviation) or associated estimates of uncertainty (e.g. confidence intervals)
☐	☒	For null hypothesis testing, the test statistic (e.g. *F*, *t*, *r*) with confidence intervals, effect sizes, degrees of freedom and *P* value noted<br>*Give P values as exact values whenever suitable.*
☒	☐	For Bayesian analysis, information on the choice of priors and Markov chain Monte Carlo settings
☒	☐	For hierarchical and complex designs, identification of the appropriate level for tests and full reporting of outcomes
☒	☐	Estimates of effect sizes (e.g. Cohen's *d*, Pearson's *r*), indicating how they were calculated

Our web collection on statistics for biologists contains articles on many of the points above.

Software and code

Policy information about availability of computer code

Data collection	Flow cytometry: FACSDiva 10.5.3<br>Laser scanning confocal microscopy: OLYMPUS FLUOVIEW Ver.1.7a<br>Transmission electron microscope: 832 SC1000, Gatan, Warrendale, PA
Data analysis	GraphPad Prism 9 was used to perform general statistical analysis.<br>Flow Cytometry: Flow Jo V10.

For manuscripts utilizing custom algorithms or software that are central to the research but not yet described in published literature, software must be made available to editors and reviewers. We strongly encourage code deposition in a community repository (e.g. GitHub). See the Nature Research guidelines for submitting code & software for further information.

Data

Policy information about availability of data

All manuscripts must include a data availability statement. This statement should provide the following information, where applicable:
- Accession codes, unique identifiers, or web links for publicly available datasets
- A list of figures that have associated raw data
- A description of any restrictions on data availability

Source data for figures are provided with the paper

Field-specific reporting

Please select the one below that is the best fit for your research. If you are not sure, read the appropriate sections before making your selection.

- [x] Life sciences
- [ ] Behavioural & social sciences
- [ ] Ecological, evolutionary & environmental sciences

For a reference copy of the document with all sections, see nature.com/documents/nr-reporting-summary-flat.pdf

Life sciences study design

All studies must disclose on these points even when the disclosure is negative.

Sample size	All specific sample sizes are mentioned in each figure or figure legend. No statistical tests were performed for pre-determination of sample size.
Data exclusions	No exclusions of any data for the reported results.
Replication	Number of replication for each experiment is described in the method section or corresponding figure legends. And, all replications surpport the same conclusion.
Randomization	The randomization is not relevant to this study because the objective of this study is a cohort of HFRS patients and healthy donors, which is not involved man-made interference.
Blinding	All blood samples were blindly coded to ensure anonymity of the patients or healthy donors.

Reporting for specific materials, systems and methods

We require information from authors about some types of materials, experimental systems and methods used in many studies. Here, indicate whether each material, system or method listed is relevant to your study. If you are not sure if a list item applies to your research, read the appropriate section before selecting a response.

Materials & experimental systems

n/a	Involved in the study	
[ ]	[x]	Antibodies
[ ]	[x]	Eukaryotic cell lines
[x]	[ ]	Palaeontology and archaeology
[x]	[ ]	Animals and other organisms
[ ]	[x]	Human research participants
[x]	[ ]	Clinical data
[x]	[ ]	Dual use research of concern

Methods

n/a	Involved in the study	
[x]	[ ]	ChIP-seq
[ ]	[x]	Flow cytometry
[x]	[ ]	MRI-based neuroimaging

Antibodies

Antibodies used

The following antibodies were generated by BioLegend (San Diego, CA, USA):
APC/Cyanine7 anti-human CD3 Antibody (Cat. 300318), 1:100 (Flow Cyt)
PE/Cyanine7 anti-human CD8 Antibody (Cat. 344712), 1:100 (Flow Cyt)
PE/Cyanine5 anti-human CD4 Antibody (Cat.317412), 1:100 (Flow Cyt)
APC/Cyanine7 mouse IgG2a, κ (Cat. 402206), 1:100 (Flow Cyt)
PE/Cyanine7 mouse IgG1, κ (Cat. 400126), 1:100 (Flow Cyt)
PE/Cyanine5 mouse IgG2b, κ (Cat. 402206), 1:100 (Flow Cyt)
FITC mouse IgG1, κ (Cat.400108), 1:100 (Flow Cyt)

The following antibodies were generated by Abcam (Cambridge, MA, USA):
Rabbit monoclonal anti-human CD4 antibody (ab133616), 1:200 (ICC/IF)
Rabbit monoclonal anti-human CD8 antibody (ab93278), 1:200 (ICC/IF)

The following antibodies were generated by GeneTex (San Antonio, TX, USA):
Rabbit IgG antibody (DyLight594) (GTX213110-05)
Mouse IgG antibody (DyLight488) (GTX213111-04)

The following antibody was generated by our laboratory:
The specific mAb 1A8 against HTNV-NP, 1:100 (Flow Cyt), 1:500 (ICC/IF)
For flow cytometry, mAb 1A8 was directly labeled with FITC (fluorescein isothiocyanate) following the protocol of a commercial kit

developed for primary antibody labeling (Lightning-Link® Antibody Labeling Kits, Innova Biosciences).

Validation	APC/Cyanine7 anti-human CD3 Antibody (Cat. 300318)<br>Host species: mouse; Application: Flow Cytometry; Manufacturer's web site: https://www.biolegend.com/en-us/products/apc-cyanine7-anti-human-cd3-antibody-1912?GroupID=GROUP28<br>PE/Cyanine7 anti-human CD8 Antibody (Cat. 344712)<br>Host species: mouse; Application: Flow Cytometry; Manufacturer's web site: https://www.biolegend.com/en-us/products/pe-cyanine7-anti-human-cd8-antibody-6390?GroupID=GROUP28<br>PE/Cyanine5 anti-human CD4 Antibody (Cat.317412)<br>Host species: mouse; Application: Flow Cytometry; Manufacturer's web site: https://www.biolegend.com/en-us/products/pe-cyanine5-anti-human-cd4-antibody-3655?GroupID=GROUP28<br>APC/Cyanine7 mouse IgG2a, κ (Cat. 402206)<br>Host species: mouse; Application: Flow Cytometry; Manufacturer's web site: https://www.biolegend.com/en-us/products/apc-cyanine7-mouse-igg2a-kappa-isotype-ctrl-1923?GroupID=GROUP29<br>PE/Cyanine7 mouse IgG1, κ (Cat. 400126)<br>Host species: mouse; Application: Flow Cytometry; Manufacturer's web site: https://www.biolegend.com/en-us/products/pe-cyanine7-mouse-igg1-kappa-isotype-ctrl-1926?GroupID=GROUP29<br>PE/Cyanine5 mouse IgG2b, κ (Cat. 402206)<br>Host species: mouse; Application: Flow Cytometry; Manufacturer's web site: https://www.biolegend.com/en-us/products/pe-cyanine5-rat-igg2b-kappa-isotype-ctrl-1857?GroupID=GROUP29<br>FITC mouse IgG1, κ (Cat.400108)<br>Host species: mouse; Application: Flow Cytometry; Manufacturer's web site: https://www.biolegend.com/en-us/products/fitc-mouse-igg1-kappa-isotype-ctrl-1406?GroupID=GROUP29<br>Rabbit monoclonal anti-human CD4 antibody (ab133616)<br>Host species: Rabbit; Suitable for: ICC, WB, IHC-P, Flow Cyt; Manufacturer's web site: https://www.abcam.com/CD4-antibody-EPR6855-ab133616.html<br>Rabbit monoclonal anti-human CD8 antibody (ab93278)<br>Host species: Rabbit; Suitable for: WB, IHC-P, ICC; Manufacturer's web site: https://www.abcam.com/cd8-alpha-antibody-ep1150y-ab93278.html<br>Rabbit IgG antibody (DyLight594) (GTX213110-05)<br>Host species: Goat; Suitable for: WB, ICC/IF, IHC-P, IHC-Fr, FACS; Manufacturer's web site: https://www.genetex.cn/Product/Detail/Goat-Anti-Rabbit-IgG-antibody-DyLight594/GTX213110-05<br>Mouse IgG antibody (DyLight488) (GTX213111-04)<br>Host species: Goat; Suitable for: WB, ICC/IF, IHC-P, IHC-Fr, FACS; Manufacturer's web site: https://www.genetex.cn/Product/Detail/Goat-Anti-Mouse-IgG-antibody-DyLight488/GTX213111-04<br>FITC anti-HTNV NP Antibody<br>Host species: Mouse; Suitable for: Flow Cyt; Validated in this paper.

Eukaryotic cell lines

Policy information about cell lines

Cell line source(s)	Vero E6 and A549 cells were purchased from ATCC.
Authentication	Vero E6 and A549 cells were authenticated based on our vast experiments working with these cell lines (such as cell morphology, culture condition, cell characteristics).
Mycoplasma contamination	Cells were routinely tested for mycoplasma contamination, and only negative cells were used for experiments.
Commonly misidentified lines (See ICLAC register)	None

Human research participants

Policy information about studies involving human research participants

Population characteristics	Men and women 14-79 years of age lived in China.
Recruitment	This study included 280 blood samples from 119 HFRS patients hospitalized in the Department of Infectious Diseases of the Fourth Military Medical University (Xi'an, China) from October 2017 to January 2020.Two study cohorts were established, No.1 included 158 samples from 74 patients from November 2017 to January 2018, and No.2 included 122 samples from 45 patients from November 2019 to January 2020.HTNV infection was diagnosed by serological testing according to diagnostic criteria. HTNV infection was confirmed via serological testing of immunoglobulin M (IgM) and immunoglobulin G (IgG) in serum specimens. According to the diagnostic criteria in the Prevention and Treatment Strategy of HFRS, the acute phase includes febrile, hypotensive, and oliguric stages; the convalescent phase includes diuretic and convalescent stages.To control for potential confounders, we excluded patients with autoimmune diseases, viral hepatitis, hematological diseases, diabetes, cardiovascular diseases, and other kidney or liver diseases.
Ethics oversight	This study was approved by the First Affiliated Hospital of Fourth Military Medical University IRB (KY20173166-1). Written informed consent was obtained from the patient before inclusion in the study.

Note that full information on the approval of the study protocol must also be provided in the manuscript.

Flow Cytometry

Plots

Confirm that:

- [x] The axis labels state the marker and fluorochrome used (e.g. CD4-FITC).
- [x] The axis scales are clearly visible. Include numbers along axes only for bottom left plot of group (a 'group' is an analysis of identical markers).
- [x] All plots are contour plots with outliers or pseudocolor plots.
- [x] A numerical value for number of cells or percentage (with statistics) is provided.

Methodology

Sample preparation	Fresh peripheral blood of the HFRS patients and healthy donors was used to isolate the PBMCs using density gradient centrifugation. The CD4+ and CD8+ T lymphocytes were rapidly purified from the PBMCs by magnetic cell sorting approach (Miltenyi Biotech Inc., Auburn, CA). And, 0.2 million cells were labeling with different fluorophore conjugated antibodies at 4 ℃for 40 mins, followed by two washes.
Instrument	Cells were collected using ACEA NovoCyte™ flow cytometry (ACEA Biosciences Inc. China).
Software	Flow-cytometric analysis was performed immediately with FlowJo version 10 (TreeStar).
Cell population abundance	The purity of the resulting CD4+ and CD8+ T cells was consistently > 96%, as determined by flow cytometry.
Gating strategy	Gates for determining positivity were established by using isotype controls so that 99.0% of events were negative.

- [x] Tick this box to confirm that a figure exemplifying the gating strategy is provided in the Supplementary Information.
